# Developing a Large Language Model–Based Feedback System for Case Report Writing in Rehabilitation Education: Tutorial

**DOI:** 10.2196/76924

**Published:** 2026-06-15

**Authors:** Yuuto Tonouchi, Shunsuke Nakai, Kayo Murakami, Yuki Kataoka

**Affiliations:** 1 Department of Rehabilitation Kyoto Min-iren Asukai Hospital Kyoto, Kyoto Japan; 2 Department of Occupational Therapy Faculty of Rehabilitation Morinomiya University of Medical Sciences Osaka, Osaka Japan; 3 Graduate School of Rehabilitation Science Osaka Metropolitan University Osaka, Osaka Japan; 4 Center for Postgraduate Clinical Training and Career Development Nagoya University Hospital Nagoya, Aichi Japan; 5 Center for Medical Education Graduate School of Medicine Nagoya University Nagoya, Aichi Japan; 6 Department of Healthcare Epidemiology Graduate School of Medicine and Public Health Kyoto University Kyoto, Kyoto Japan; 7 Department of Systematic Reviewers Scientific Research Works Peer Support Group (SRWS-PSG) Osaka, Osaka Japan; 8 Department of Internal Medicine Kyoto Min-iren Asukai Hospital Kyoto, Kyoto Japan

**Keywords:** large language model, education, artificial intelligence, AI, rehabilitation, case report, feedback

## Abstract

**Background:**

Novice health care staff often write case reports during early clinical training. However, many institutions lack structured feedback systems because of time constraints and instructor shortages. Large language models, a form of artificial intelligence (AI), offer new opportunities to enhance educational feedback, yet their application in clinical training requires careful design to ensure pedagogically appropriate and ethically sound outputs.

**Objective:**

This tutorial provides a practical guide for educators without programming experience to develop an AI-based feedback system using 3 accessible tools: Dify (an AI workflow platform), Slack (a messaging app), and Google Apps Script. The system balances educational quality with operational efficiency while incorporating data privacy safeguards for clinical educational content.

**Methods:**

We developed a feedback system comprising 4 AI chatbots with 2 distinct approaches: “loop-based” bots that promote clinical reasoning through iterative, comment-based feedback and “single-shot” bots for efficient proofreading and summarization tasks. The tutorial describes the system architecture; feedback design principles grounded in formative assessment theory; a step-by-step implementation guide; and data privacy safeguards, including a deidentification protocol and application programming interface–based data protection measures. To illustrate feasibility, we conducted a pilot implementation at a community care hospital from April to June 2024, involving 5 novice staff members and 5 instructors.

**Results:**

A pilot implementation at a community care hospital demonstrated that the system was feasible to deploy and operate within routine clinical education workflows. Participant feedback indicated high usability and suggested that the iterative, comment-based feedback approach supported learner engagement while also identifying areas where feedback criteria required refinement to better match institutional educational goals.

**Conclusions:**

This tutorial provides a reproducible framework for building a customized AI feedback system that combines comment-based iterative feedback with human-in-the-loop oversight and a multilayered data privacy framework. By following this guide, educators can implement an adaptive system tailored to their institutional context and clinical domain.

## Introduction

### Background

In medical education, writing is not merely a record of clinical events but a vital learning activity that supports reflection and growth. Reflective writing (RW), including the reflective elements embedded in case report writing, fosters core competencies such as clinical reasoning and professional identity formation [1,2]. However, writing becomes educationally meaningful only when feedback effectively guides learners toward explicit goals [3]. Previous studies confirm that supportive, structured, and longitudinal feedback maximizes the impact of RW [2,3].

Delivering such feedback at scale faces persistent challenges in clinical settings. Time constraints and limited instructional skills often prevent adequate feedback [1-3]. Consequently, this feedback loop frequently breaks down, hindering the professional development of novice staff.

Large language models (LLMs) have demonstrated utility for feedback, clinical reasoning, and research assistance, including health care contexts [4-6]. A recent review suggests that artificial intelligence (AI) reduces workload and improves efficiency, particularly in medical writing and administrative support [7]. These developments have increased interest in applying LLMs to educational feedback for RW.

However, realizing this potential requires purpose-built systems that go beyond general-purpose chatbot interactions, along with careful consideration of data privacy. This tutorial addresses that gap.

### Aims and Target Audience

This tutorial provides a practical guide for educators without programming experience to develop an AI-based feedback system for case report writing in clinical education. Specifically, it offers (1) a conceptual framework distinguishing custom AI systems from standard chatbot use, (2) a step-by-step implementation guide using 3 accessible tools (Dify [LangGenius, Inc]; Slack [Slack Technologies, LLC]; and Google Apps Script [GAS; Google LLC]), (3) ethical and data privacy guidelines for deploying AI in medical education settings, and (4) empirical evaluation data from a pilot implementation at a community care hospital.

The primary audience is clinical educators who supervise novice staff writing case reports, experience time pressure when providing feedback, and lack programming expertise. Although the examples and prompts presented here focus on rehabilitation settings as a working illustration, educators in other medical fields can adapt this framework by modifying the prompts to suit their specific clinical domains and educational objectives.

### Rationale for a Customized System

General-purpose chatbots (eg, a standard ChatGPT interface) pose substantial risks when used without customization in medical education. The medical domain requires specialized terminology and linguistic precision that general models may fail to process accurately [8]. Effective AI use in medical education depends on strategic prompt design; poorly constructed instructions can yield inaccurate or unsafe outputs [9]. Mastering such prompt design techniques requires technical expertise that most clinical educators do not possess [9], making accessibility a core design requirement. These considerations led us to develop a customized system rather than rely on off-the-shelf chatbot interactions.

Three requirements distinguish a custom educational system from standard chatbot use. First, standardization ensures fairness. In direct chatbot interaction, response quality depends entirely on the user’s prompting skills. Novice staff may receive suboptimal feedback simply due to limited prompting skills. A custom system uses preprogrammed instructions, known as meta-prompts (Textbox 1), to evaluate every draft against the same rubric, ensuring consistent feedback for all learners. Second, integration enables visibility and progress management. When novice staff use personal chatbot accounts, educators cannot monitor their interactions. Integrating the system into a shared workspace enables educators to transparently track progress and intervene when necessary. Third, centralized logging supports educational auditing. To improve the curriculum, educators need to identify common errors among novice staff. A custom system automatically records all interactions, generating valuable data for analyzing trends in learner performance.

**Prompts vs meta-prompts (user input vs system instructions).**

This system distinguishes between 2 types of text inputs:

1. Prompts (user input): the text written by the novice staff (eg, “Here is my draft...”).
2. Meta-prompts (system instruction): hidden instructions preset by the educator. This text contains the persona (eg, “You are a senior physical therapist”), the rubric, and formatting rules. Separating these components ensures that the artificial intelligence consistently functions as a trained instructor, regardless of what the novice staff member inputs.

## Methods

### Highlights

This section is organized as follows. First, we describe the 3-component system architecture (Dify, Slack, and GAS) and the dataflow connecting them. Second, we present the feedback design principles and the specifications of 4 educational chatbots. Third, we provide a step-by-step implementation guide for reproducing the system. Fourth, we address ethical considerations and data privacy safeguards. Finally, we describe a pilot implementation conducted at a community care hospital to assess system usability and feasibility.

### System Architecture and Dataflow

**Figure 1:**
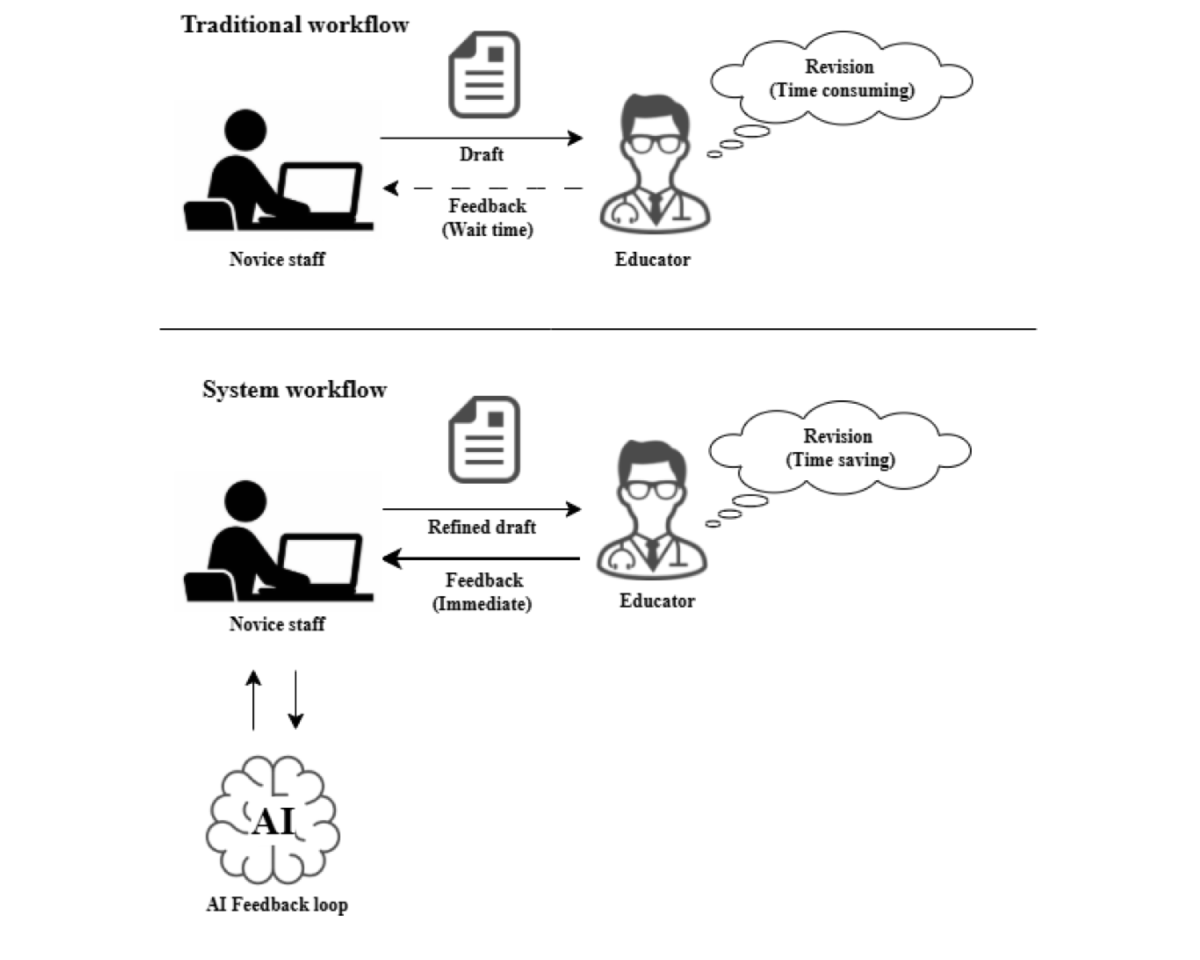
Operational flow comparison. AI: artificial intelligence.

Figure 1 illustrates the operational workflow. From the novice staff’s perspective, the system provides a straightforward interaction: the novice staff tags the relevant bot in Slack (eg, @Discussion_Bot) and submits a draft text. The system processes the request and provides specific feedback within the same thread. The novice staff then revises the text and replies to the bot again. This iterative conversation continues until the draft meets the required standards. This workflow off-loads the time-consuming revision cycle to the AI, reserving human feedback for clinical validity and professional judgment.

**Figure 2:**
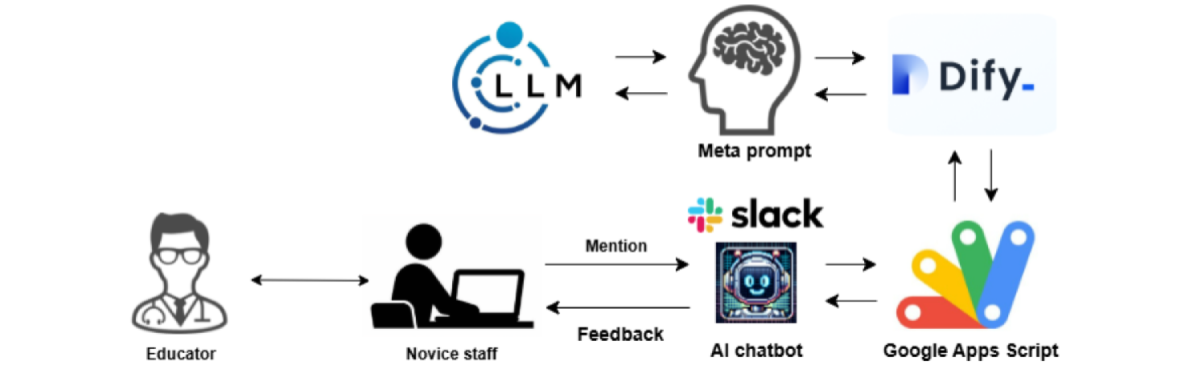
Three-component system architecture. AI: artificial intelligence. LLM: large language model.

The system architecture consists of 3 components selected to enable implementation without advanced programming knowledge (Figure 2). Programming is a well-documented barrier for health care professionals seeking to develop AI applications [10]. We therefore adopted an approach that does not require specialized programming expertise and uses the following 3 components: Dify, Slack, and GAS.

Dify [11] is an open-source LLM application development platform that serves as the system’s central processing engine. Hosted in the cloud, it receives user inputs, processes them through a predesigned workflow, and instructs the AI how to respond. Educators can build the AI’s reasoning process visually, without writing code. We selected Dify because it offered the most accessible visual interface among available open-source LLM workflow platforms, combined with built-in application programming interface (API) publishing and conversation logging. The free tier is suitable for initial testing but has limited message credits; a paid plan may be required for sustained use with multiple learners. Data sovereignty considerations are discussed in the Ethical Considerations and Data Privacy section.

Slack [12] serves as the interactive chat interface for novice staff. Its private channel structure enables educators to monitor all novice staff-AI interactions in real time and to communicate with novice staff within the same workspace. The free tier limits message history retention, which we addressed through automatic logging to Google Sheets via GAS.

GAS functions as the technical bridge linking Slack and Dify, securely relaying messages between the 2 platforms. We chose GAS for its native integration with Google Workspace, enabling automatic logging to Google Sheets and administrator error notifications without additional infrastructure. Although Dify provides a built-in Slack plug-in for direct integration, it does not support the logging and oversight functions considered essential for educational management. When a novice staff sends a message on Slack, the data travels through GAS to reach Dify, and the response returns along the same path. Every interaction is automatically logged, visible to educators, and monitored for errors.

**Figure 3:**
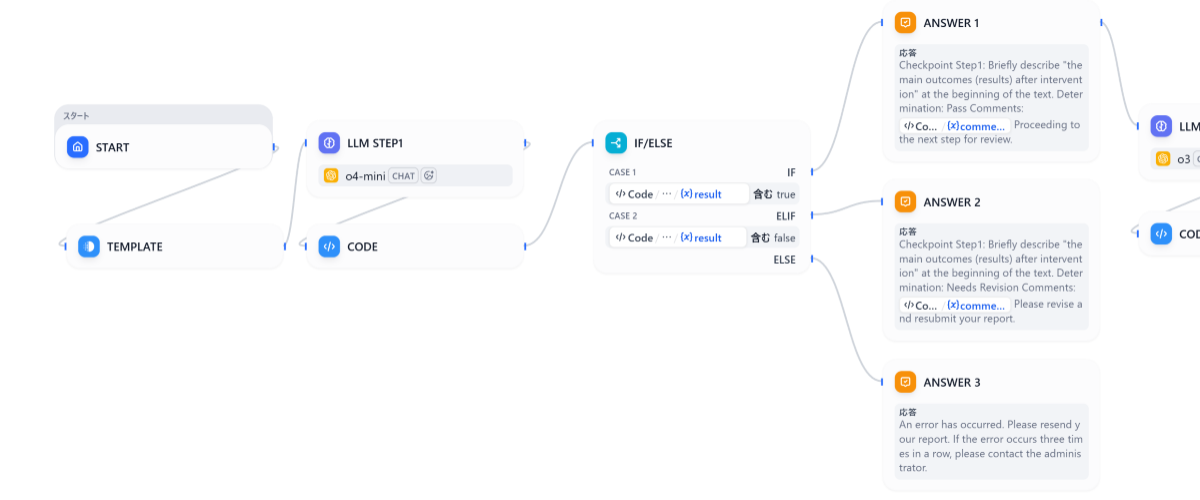
Dify Chatflow for the conditional workflow. LLM: large language model.

A key design feature is the iterative pass or revise loop implemented within Dify’s Chatflow functionality (Figure 3). Unlike a simple chatbot that answers a question once, effective education requires iterative engagement [3,13]. A recent Association for Medical Education in Europe guide indicates that continuous, formative feedback aligned with explicit learning objectives develops competencies more effectively than one-time summative evaluation [14]. The system, therefore, implements a conditional workflow:

- Analyze: the system evaluates the novice staff’s draft against the defined rubric.
- Decide the following:
  - If the draft meets the criteria, the system outputs “pass” and allows the novice staff to proceed.
  - If not, the system outputs “revise,” provides targeted comments, and awaits the corrected draft.
- Repeat: this mechanism prevents novice staff from overlooking feedback and promotes iterative learning.

### Feedback Design and Bot Specifications

#### Overview

The feedback design rests on 2 complementary principles: comment-based feedback and the human-in-the-loop approach.

Comment-based feedback prioritizes written comments over direct correction. Research suggests that comments produce greater gains in writing proficiency than simple error location or direct correction [13] because comments compel learners to actively process information and revise their own work [14]. The system thus functions as an always-available virtual instructor, serving as a strict gatekeeper prior to human review. By off-loading the repetitive task of checking logical consistency and structure to the AI, educators can focus on the final clinical assessment.

The human-in-the-loop approach ensures that educators retain final decision-making authority; AI serves only as a support tool. The AI focuses on overall structure, logical consistency, and writing style, while avoiding specific clinical judgments to mitigate the risk of hallucinations (ie, plausible but incorrect medical advice). This system does not evaluate clinical validity or refine medical discussions. Qualified educators must provide professional feedback on these aspects, and novice staff should treat AI feedback as suggestions for improving writing structure and logic, not as clinical guidance.

On the basis of these principles, the system provides 4 distinct chatbots tailored to the report writing process (Table 1; refer to Multimedia Appendix 1 for detailed meta-prompts.) The system uses 2 distinct feedback approaches: a “loop-based” approach for iterative revision and a “single-shot” approach for efficient one-time processing.

**Table 1:** Specifications and logic of the 4 chatbots.

Chatbot name and type	Primary function	Evaluation criteria or prompt elements
Integration and interpretation bot (loop-based feedback)	Verifies clinical reasoning and checks consistency between assessment, problems, and goals.	Passing criteria (checkpoints) Description of the clinical overview Clarification of focal points and rationale Identification of specific clinical problems Alignment of assessment data with identified problems Formulation of treatment plan and prognosis
Discussion bot (loop-based feedback)	Evaluates argumentation and checks if the argument logically supports the conclusion based on evidence.	Passing criteria (checkpoints) Summary of key intervention outcomes Integration of relevant literature and guidelines Comparative analysis with previous studies and original conclusions
Proofreading bot (single-shot output)	Enhances text quality and acts as a domain-specific AI^a^ editor to refine style and grammar for academic submission.	Prompt elements Detect and correct typographical and grammatical errors Standardize tone to formal academic style Refine expressions for clarity and conciseness Ensure logical consistency and readability Strengthen paragraph coherence
Abstract summarization bot (single-shot output)	Generates structured summaries and condenses the case report into a format suitable for conference or journal abstract submission.	Output specifications Generate a structured abstract text Propose 2 candidate titles Select 3 relevant keywords Provide the final summarized text

^a^AI: artificial intelligence.

#### Loop-Based Chatbots

The 2 loop-based chatbots promote clinical reasoning through iterative, comment-based feedback:

- Integration and interpretation bot: verifies clinical reasoning by checking the logical consistency between assessment results, the problem list, and goal setting. The bot prompts revisions until all logical checkpoints are met.
- Discussion bot: evaluates whether the argument aligns with the interpretation and logically supports the conclusion. The bot prompts revisions until all checkpoints are satisfied.

#### Single-Shot Chatbots

The 2 single-shot chatbots perform efficient, single-pass tasks:

- Proofreading bot: corrects grammatical errors and improves clarity without altering the clinical meaning. It generates a polished draft in a single interaction.
- Abstract summarization bot: produces a concise summary of the completed case report in a format suitable for conference or journal submission.

### Step-By-Step Implementation

#### Overview

This guide enables educators to build the system by following step-by-step instructions and copying the provided resources; no complex coding is required. The following subsections outline the role of each component and the key actions involved. Detailed instructions, including specific platform operations, configuration values, and troubleshooting guidance, are provided in Multimedia Appendix 2.

#### Prerequisites: Accounts and API Keys

Ensure the following are ready before starting:

- Dify account: (cloud version or self-hosted)
- Slack workspace: (educators need permission to create apps and add bots)
- Google account: (to access Google Drive and Sheets)
- API keys: an API key from an LLM provider (eg, OpenAI API key or Anthropic API key; Textbox 2).

**Understanding application programming interface (APIs) and API keys.**

- What is an API? An API is a set of rules that allows different software systems to communicate. It acts as a standardized bridge, sending a digital request from your system to the artificial intelligence (AI) service and returning the response. This allows your application to use powerful external AI capabilities without hosting the underlying technology locally.
- What is an API key? An API key is a unique string of characters that acts as a digital ID card (authentication token). When Dify sends a request to a large language model provider (such as OpenAI), it uses this key to verify the user’s identity and link use to a specific billing account. Warning: never share this key publicly. If compromised, unauthorized users may incur charges against your account.

#### Setting Up Dify

Dify serves as the AI processing engine. Implementation involves creating a new Chatflow-type application, importing the feedback workflow via the domain-specific language file (Multimedia Appendix 3), registering the LLM API key, and adjusting the meta-prompts to suit the target clinical domain. Once published, Dify generates an API key for use in subsequent integration steps.

#### Creating the Slack Application

After configuring Dify, the next step is to create the bot account in Slack. This involves registering a new app in the Slack API console, assigning the minimum necessary permissions (eg, reading messages, sending replies), and installing the bot in the target workspace. The resulting bot token is required for the integration step.

#### Configuring GAS

Dify handles the AI logic, and Slack provides the chat interface, but the 2 platforms cannot communicate directly. GAS serves as the bridge connecting them. Implementation involves creating a GAS project, deploying the relay script (Multimedia Appendix 4), and securely storing the API credentials from the previous steps as script properties. The script is then deployed as a web application so that Slack can send messages to it.

#### Final Configuration and Verification

The final step links all 3 components and verifies system function. Configuring Slack’s event subscriptions feature to forward messages to the GAS end point completes the communication loop. A test message sent in Slack should trigger the full pipeline: Slack receives the message, GAS relays it to Dify, the LLM generates feedback, and the response returns to Slack while being logged in Google Sheets.

### Data Privacy

Because this system transmits case report content through multiple external platforms, safeguarding protected health information (PHI) is essential. This section describes the data protection measures organized into 3 categories: administrative, technical, and supervisory safeguards.

#### Administrative Safeguards

Before submitting any draft to the system, novice staff must deidentify all patient information following the anonymization standards recommended by the International Committee of Medical Journal Editors [15]. The deidentification protocol involves 2 steps:

1. Remove all direct identifiers: patient names, medical record numbers, dates of birth, addresses, and contact information.
2. Generalize quasi-identifiers to prevent reidentification through combination: convert specific dates to relative timelines (eg, “Day 1 of admission” rather than a calendar date), express exact ages in decade ranges (eg, “a patient in their 80s”), and replace facility names with generic descriptors (eg, “a community rehabilitation hospital”).

In the pilot implementation, we provided deidentification training to all participants prior to system use (refer to the Ethical Considerations section). Each novice staff member received individual instruction on the protocol, supplemented by a short instructional video demonstrating the 2-step process with concrete examples from rehabilitation case reports. We reinforced compliance through ongoing supervision during the pilot period.

#### Technical Safeguards

All communication with LLMs in this system occurs exclusively through the API, not through consumer-facing chat interfaces such as ChatGPT (OpenAI) or Claude.ai (Anthropic). This architectural choice provides a contractual safeguard: both OpenAI and Anthropic explicitly state in their API terms of service that data submitted through the API are not used to train or improve their models [16,17]. Both OpenAI and Anthropic retain API data for up to 30 days solely for abuse monitoring purposes, after which it is automatically deleted. Because data submitted to LLM providers cannot be recalled during this retention window, deidentification prior to submission constitutes the critical safeguard.

**Figure 4:**
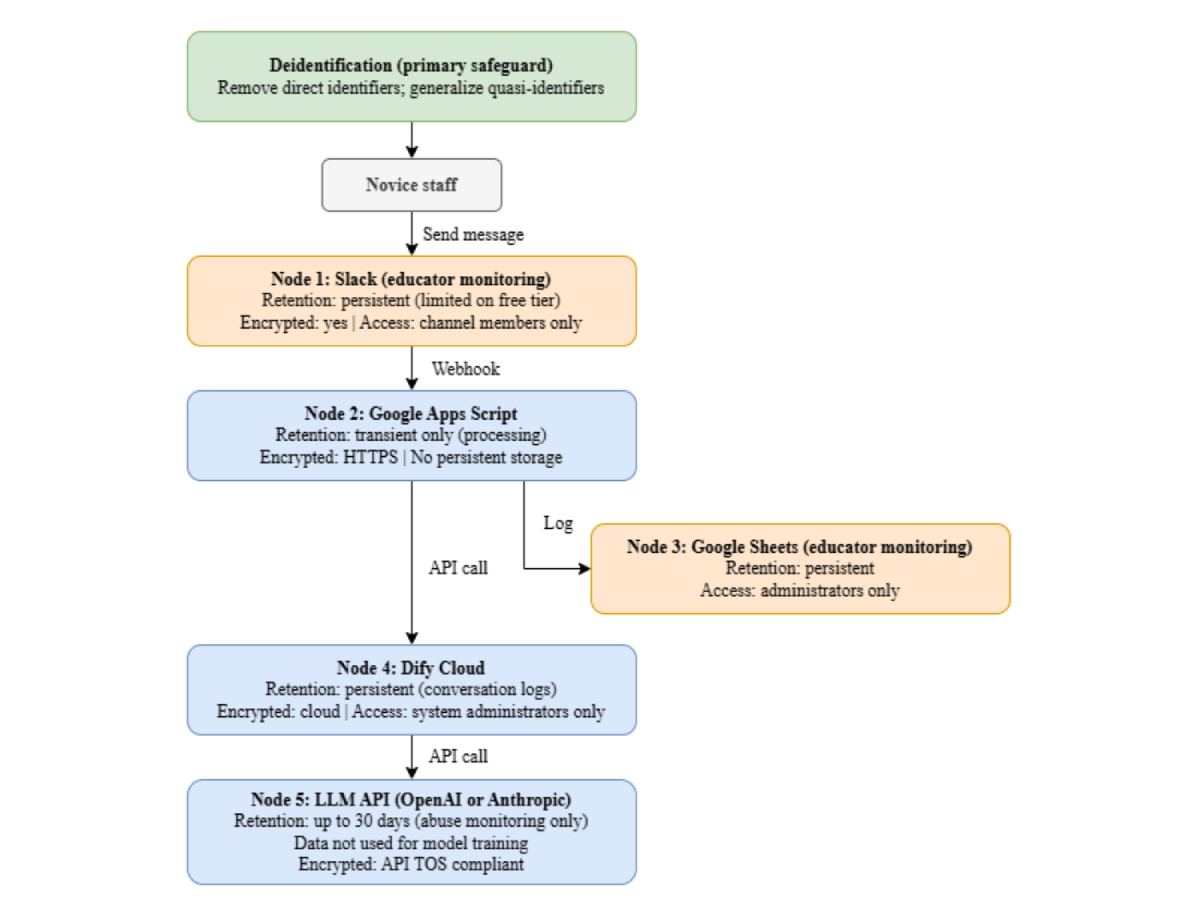
Dataflow and privacy safeguards across the 5 system nodes. API: application programming interface; LLM: large language model.

To provide a transparent risk assessment, we trace the complete data path (Figure 4). When a novice staff submits a message in Slack, the data traverses 5 external nodes before a response is returned:

1. Slack servers: Slack stores messages with encryption; access is restricted to invited channel members.
2. GAS: GAS processes data transiently during relay without persistent storage.
3. Google Sheets: conversation logs are persistently stored for educational analysis, with access restricted to designated administrators.
4. Dify Cloud: conversation logs are retained within the platform and are accessible only to the system administrator.
5. LLM API end point (OpenAI or Anthropic): the LLM provider processes data and retains it for up to 30 days under the nontraining policies described above.

For institutions requiring full data sovereignty, Dify offers a self-hosted deployment option that retains all data within the institution’s own infrastructure, though this requires server infrastructure and technical expertise.

#### Real-Time Oversight and Incident Response

The system architecture supports real-time oversight. Because all interactions occur in shared Slack channels, educators can continuously monitor both novice staff submissions and AI responses, enabling immediate detection of any inadvertent PHI disclosure. The Google Sheets log provides a persistent, searchable record for periodic auditing. If PHI is inadvertently submitted, educators can take the following remediation steps:

- Delete the PHI-containing message from Slack.
- Remove the corresponding row from the Google Sheets log.
- Purge the conversation log from the Dify platform.
- The LLM provider automatically deletes data within the 30-day retention window.

The current system does not include automated PHI detection prior to submission; deidentification relies on the administrative safeguards described above. We identify the development of automated presubmission screening as a priority for future implementations (refer to the limitations and future directions section).

#### Transparency, Consent, and Scope of AI Feedback

Transparency is foundational to ethical AI adoption. Before introducing the system, educators should clearly explain to novice staff that they are communicating with an AI system and how their data will be processed. Educators should also define the system as an educational support tool for facilitating the drafting process, rather than a replacement for human supervision or a formal assessment of clinical capabilities.

To manage the risk of hallucinations, the system uses 2 strategies. First, the AI’s feedback includes a disclaimer: “AI can make mistakes. Always verify medical facts with your supervisor.” Second, the AI’s role is limited to structural and logical feedback rather than clinical diagnosis, reducing the risk of harmful errors.

### Ethical Considerations

The ethics committee of Kyoto Min-iren Asukai Hospital approved this pilot evaluation (2024-0502). All participating staff members received verbal and written explanations of the study’s purpose and data handling procedures, and provided written informed consent. We explained deidentification requirements to all novice staff members individually and reinforced them through an instructional video prior to system use. For past case reports used in meta-prompt tuning, we implemented an opt-out procedure. Participants received no financial compensation.

### Iterative Development Process

The current Dify-based system evolved through iterative refinement of an earlier prototype. The initial version used a single-prompt chatbot approach. Early testing revealed 2 challenges: a ceiling effect in which most novice staff passed all checkpoints on their first submission, indicating that the meta-prompts were too lenient for meaningful learning, and declining instruction adherence as prompt length increased. These issues motivated the transition to the Dify-based Chatflow architecture, which evaluates checkpoints sequentially and allows educators to adjust passing criteria through its visual interface.

Some instructors also expressed concern that overreliance on AI feedback could diminish clinical reasoning skills. We therefore designed the system to limit AI feedback to structural and logical aspects and to prompt learners to reconsider their own reasoning rather than providing corrected example text.

### Pilot Implementation

To inform the design and iterative refinement of this tutorial, we conducted a single-center pilot implementation at Kyoto Min-iren Asukai Hospital. This pilot used a prototype version with direct Slack-GAS-AI integration prior to the introduction of Dify. Because the user interface (Slack) and the fundamental feedback logic (iterative revisions) remained identical, the usability data are directly applicable to the Dify-based system.

Five novice rehabilitation staff members (3 physiotherapists and 2 occupational therapists) who joined in April 2024 and 5 educational instructors participated. The pilot ran from April to June 2024. Prior to this pilot, the facility had not used Slack for official communication; thus, this pilot represented the participants’ first experience with the platform. During the pilot, participants tagged the specific bot (eg, “@Integration_Bot”) in Slack to initiate iterative feedback on structure and logic. Once the content was finalized, they used the “@Proofreading_Bot” for grammatical corrections before submitting the final draft to their human supervisors.

We collected data through 2 methods. First, postimplementation surveys via Google Forms assessed the system’s usability and acceptance. Novice staff completed the System Usability Scale [18] and rated the appropriateness of the AI feedback content. The System Usability Scale is a standardized 10-item questionnaire for measuring system usability. Scores above 68 indicate above-average usability, and scores above 85 indicate excellent usability. Instructors assessed changes in instructional efficiency and feedback appropriateness. Second, we analyzed use logs from the Slack-linked spreadsheet, including use frequency, revision counts per task, and the content of feedback.

## Results

This section describes the pilot implementation experience, which informed the iterative refinement and final design of this tutorial.

The postimplementation survey response rate was 100% (5/5) for both instructors and novice staff. Detailed survey results are provided in Multimedia Appendix 5.

Novice staff found the system accessible, with a median System Usability Scale score of 90 (range 70-95), confirming that the approach lowered the barrier to adoption. All 5 staff members reported that the AI feedback was helpful for their learning (median 5, range 4-5). Instructor feedback identified areas where the tutorial framework requires further customization: while 80% (4/5) of staff members anticipated a reduction in future instructional burden (median 4, range 1-5), ratings for the appropriateness of AI feedback content were moderate (median 3, range 1-4), suggesting that the balance between structural feedback and clinical depth needs institution-specific calibration.

Log analysis confirmed that all 5 novice staff members actively used the system. Typically, they used the integration and interpretation bot 1 to 2 times and the proofreading bot once per report.

Qualitative feedback from novice staff highlighted the psychological benefits of the system, with comments such as “My mental burden is reduced because I can get feedback before showing it to a human”. Instructors acknowledged efficiency gains, noting the ability to “focus on specialized feedback”. However, some instructors expressed concern that reliance on the system could lead to a decline in writing skills and clinical reasoning abilities.

## Discussion

### Principal Findings

This tutorial presents a practical framework for building an LLM-based feedback system for case report writing in clinical education. The principal contribution is demonstrating that educators can construct a customized AI feedback system by combining 3 accessible tools (Dify, Slack, and GAS) while maintaining educational quality and data privacy.

The system embodies 2 key design principles. First, comment-based iterative feedback encourages active revision rather than passive correction, preserving the cognitive effort essential for developing clinical reasoning skills [3,13,14]. Second, the human-in-the-loop principle limits AI to structural and logical evaluation, reserving clinical validity assessment for qualified educators and mitigating the risk of unwarranted clinical authority [19].

The pilot implementation experience supported the design decisions underlying this tutorial. In particular, the transition from the prototype to the Dify-based architecture was directly motivated by 2 challenges observed during early testing: a ceiling effect indicating that initial meta-prompts were insufficiently stringent and declining instruction adherence as prompt complexity increased. These observations illustrate a core advantage of the framework presented here: educators can iteratively adjust passing criteria and feedback logic through Dify’s visual interface, enabling continuous adaptation without programming expertise.

### Comparison With Prior Work

To our knowledge, few studies have provided practical, reproducible guidance for clinical educators to build customized LLM-based feedback systems. Systematic reviews of LLMs in medical education have predominantly identified studies evaluating LLM performance on examinations and clinical knowledge assessments, with very few reporting empirical implementation research [20,21]. Practical programming-free AI development guides in health care have focused on other domains, such as image classification [10], and exploratory studies in adjacent fields have examined similar accessible tools for providing formative feedback on student writing [22]. A step-by-step guide for building an LLM-based formative feedback system for clinical writing education has not been described. This tutorial addresses that gap.

The design principle of delegating structural and logical review to AI while reserving clinical judgment for educators reflects an emerging model in which AI supports, rather than replaces, expert judgment [19,20]. Prior studies on the use of AI in education have reported reductions in teachers’ workload and stress, along with improvements in instructional efficiency [23,24]. The pilot findings were consistent: instructors reported the ability to “focus on specialized feedback” rather than routine text review. This division of labor may offer a scalable approach to feedback delivery in resource-constrained educational settings.

Accessibility was a key design consideration. Prior research indicates that AI-based educational tools positively influence learning efficiency and cognitive motivation [24,25]. The pilot results supported this, with high usability ratings achieved even among staff with no prior experience using the chat platform.

This tutorial demonstrates that rubric-based LLM evaluation can be customized for domain-specific educational purposes without programming expertise. Although the potential of AI as a writing support tool is well documented [26,27] and LLMs can reliably evaluate clinical writing against structured rubrics [28], these capabilities have typically required programming expertise, limiting accessibility for clinical educators.

Overreliance on AI feedback poses recognized risks, including cognitive deskilling and diminished independent reasoning [29,30]. A recent scoping review of ethical challenges in AI-assisted medical education underscores the need for hybrid learning models that balance AI assistance with traditional teaching [19]. This tutorial addresses these concerns through the human-in-the-loop principle: the system limits AI feedback to structural and logical aspects, and all AI-generated comments include disclaimers directing novice staff to verify content with their supervisors.

### Limitations and Future Directions

This tutorial and its pilot evaluation have several limitations. First, the small sample size (5 novice staff and 5 instructors) limits causal inference. Second, as a single-center pilot, future research should involve multicenter collaborative studies. Third, the outcomes relied on subjective questionnaires. Future studies should analyze quantitative data such as the duration of instructor guidance and use independent reviewers to evaluate report quality objectively. Fourth, although we provided deidentification training through individual instruction and an instructional video, the pilot did not include an automated presubmission PHI detection mechanism. Recent studies have demonstrated that LLM-based systems can analyze conversation data while preserving privacy [31].

No incidents of PHI disclosure were identified during the pilot period, suggesting that the current approach, which aligns with standard security practices for cloud-based services, was sufficient within this controlled setting. However, future implementations should strengthen compliance through additional safeguards: supervisor-led presubmission review, prioritization of hypothetical cases during early adoption, and development of automated PHI detection systems. In contexts requiring stricter data governance, locally hosted LLMs may also be considered.

Two broader considerations apply to the adoption of this approach. The system relies on cloud-based services, which may not meet the data sovereignty requirements of all institutions. Additionally, because LLM capabilities and API policies evolve rapidly, educators should monitor LLM provider updates regarding data handling practices and model performance. Furthermore, while this tutorial provides detailed implementation instructions and reproducible resources, the step-by-step guide has not yet been independently tested by educators outside the development team.

Future directions include broader validation of the tutorial’s reproducibility across diverse clinical settings, development of automated PHI detection as a presubmission safeguard, and investigation of the system’s long-term impact on writing proficiency and clinical reasoning development.

### Conclusions

This tutorial provides a reproducible guide for building a case report feedback system using Dify and Slack without programming expertise. The architecture enables educators to fine-tune AI logic through visual interfaces, separating AI reasoning from software code. The tutorial also addresses data privacy considerations for deploying AI systems that handle clinical educational content. By emphasizing human oversight at every stage and iterative refinement of feedback criteria, this framework supports educators in adapting the system to their institutional context and evolving educational needs.

## Appendix

Multimedia Appendix 1 Meta-prompts used in the system.

Multimedia Appendix 2 Step-by-step implementation guide.

Multimedia Appendix 3 Dify domain-specific language file (importable file for reproduction).

Multimedia Appendix 4 Google Apps Script code.

Multimedia Appendix 5 Detailed questionnaire results.

## Abbreviations

AI: artificial intelligence
API: application programming interface
GAS: Google Apps Script
LLM: large language model
PHI: protected health information
RW: reflective writing

## Data Availability

The data supporting the findings of this work, including the developed meta-prompts and codes, are available in Multimedia Appendices 1 and 3-5. All other data are available upon request from the corresponding author.

## References

[1] Artioli G, Deiana L, De Vincenzo F, Raucci M, Amaducci G, Bassi MC, Di Leo S, Hayter M, Ghirotto L (2021). Health professionals and students' experiences of reflective writing in learning: a qualitative meta-synthesis. BMC Med Educ.

[2] Lim JY, Ong SY, Ng CY, Chan KL, Wu SY, So WZ, Tey GJ, Lam YX, Gao NL, Lim YX, Tay RY, Leong IT, Rahman ND, Chiam M, Lim C, Phua GL, Murugam V, Ong EK, Krishna LK (2023). A systematic scoping review of reflective writing in medical education. BMC Med Educ.

[3] Burgess A, van Diggele C, Roberts C, Mellis C (2020). Feedback in the clinical setting. BMC Med Educ.

[4] Dai W, Lin J, Jin H, Li T, Tsai YS, Gašević D (2023). Can large language models provide feedback to students? A case study on ChatGPT. Proceedings of the 2023 IEEE International Conference on Advanced Learning Technologies.

[5] Liang W, Zhang Y, Cao H, Wang B, Ding DY, Yang X, Vodrahalli K, He S, Smith DS, Yin Y, McFarland DA, Zou J (2024). Can large language models provide useful feedback on research papers? A large-scale empirical analysis. NEJM AI.

[6] Rossettini G, Cook C, Palese A, Pillastrini P, Turolla A (2023). Pros and cons of using artificial intelligence chatbots for musculoskeletal rehabilitation management. J Orthop Sports Phys Ther.

[7] Meng X, Yan X, Zhang K, Liu D, Cui X, Yang Y, Zhang M, Cao C, Wang J, Wang X, Gao J, Wang YG, Ji JM, Qiu Z, Li M, Qian C, Guo T, Ma S, Wang Z, Guo Z, Lei Y, Shao C, Wang W, Fan H, Tang YD (2024). The application of large language models in medicine: a scoping review. iScience.

[8] Heston TF, Khun C (2023). Prompt engineering in medical education. Int Med Educ.

[9] Zaghir J, Naguib M, Bjelogrlic M, Névéol A, Tannier X, Lovis C (2024). Prompt engineering paradigms for medical applications: scoping review. J Med Internet Res.

[10] Hoseini SS, Dewar R (2024). Empowering healthcare professionals with no-code artificial intelligence platforms for model development, a practical demonstration for pathology. Discoveries (Craiova).

[11] Dify.

[12] Slack.

[13] Biber D, Nekrasova T, Horn B (2014). The effectiveness of feedback for L1-english and L2-writing development: a meta-analysis. ETS Res Rep Ser.

[14] Lipnevich AA, Mattern K, Feddock C (2025). Formative assessment and feedback in medical education: a practical guide: AMEE guide no. 189. Med Teach.

[15] International Committee of Medical Journal Editors.

[16] Data controls in the OpenAI platform. OpenAI.

[17] Is my data used for model training?. Anthropic.

[18] Brooke J (1996). SUS: a 'quick and dirty' usability scale. Usability Evaluation in Industry.

[19] Li X, Yan X, Lai H (2025). The ethical challenges in the integration of artificial intelligence and large language models in medical education: a scoping review. PLoS One.

[20] Lucas HC, Upperman JS, Robinson JR (2024). A systematic review of large language models and their implications in medical education. Med Educ.

[21] Aster A, Laupichler MC, Rockwell-Kollmann T, Masala G, Bala E, Raupach T (2024). ChatGPT and other large language models in medical education - scoping literature review. Med Sci Educ.

[22] Venter J, Coetzee SA, Schmulian A (2024). Exploring the use of artificial intelligence (AI) in the delivery of effective feedback. Assess Eval High Educ.

[23] Younas A, Subramanian KP, Haziazi MA, Hussainy SS, Kindi AN (2023). A review on implementation of artificial intelligence in education. Int J Res Innov Soc Sci.

[24] Kasneci E, Sessler K, Küchemann S, Bannert M, Dementieva D, Fischer F, Gasser U, Groh G, Günnemann S, Hüllermeier E, Krusche S, Kutyniok G, Michaeli T, Nerdel C, Pfeffer J, Poquet O, Sailer M, Schmidt A, Seidel T, Stadler M, Weller J, Kuhn J, Kasneci G (2023). ChatGPT for good? On opportunities and challenges of large language models for education. Learn Individ Differ.

[25] Meyer J, Jansen T, Schiller R, Liebenow LW, Steinbach M, Horbach A, Fleckenstein J (2024). Using LLMs to bring evidence-based feedback into the classroom: AI-generated feedback increases secondary students’ text revision, motivation, and positive emotions. Comput Educ Artif Intell.

[26] Ho WL, Koussayer B, Sujka J (2023). ChatGPT: friend or foe in medical writing? An example of how ChatGPT can be utilized in writing case reports. Surg Pract Sci.

[27] Abd-Alrazaq A, AlSaad R, Alhuwail D, Ahmed A, Healy PM, Latifi S, Aziz S, Damseh R, Alabed Alrazak S, Sheikh J (2023). Large language models in medical education: opportunities, challenges, and future directions. JMIR Med Educ.

[28] Burke HB, Hoang A, Lopreiato JO, King H, Hemmer P, Montgomery M, Gagarin V (2024). Assessing the ability of a large language model to score free-text medical student clinical notes: quantitative study. JMIR Med Educ.

[29] Choudhury A, Chaudhry Z (2024). Large language models and user trust: consequence of self-referential learning loop and the deskilling of health care professionals. J Med Internet Res.

[30] Izquierdo-Condoy JS, Arias-Intriago M, Tello-De-la-Torre A, Busch F, Ortiz-Prado E (2025). Generative artificial intelligence in medical education: enhancing critical thinking or undermining cognitive autonomy?. J Med Internet Res.

[31] Tamkin A, McCain M, Handa K, Durmus E, Lovitt L, Rathi A, Huang S, Mountfield A, Hong J, Ritchie S, Stern M, Clarke B, Goldberg L, Sumers TR, Mueller J, McEachen W, Mitchell W, Carter S, Clark J, Kaplan J, Ganguli D Clio: privacy-preserving insights into real-world AI use. arXiv.

[32] Google Gemini.

