# Supplementary material for: Developing a Large Language Model–Based Feedback System for Case Report Writing in Rehabilitation Education: Tutorial
Source: JMIR Med Educ. 2026 Jun 15;12:e76924. doi: 10.2196/76924 (PMC13315997; doi:10.2196/76924)
Supplement: Multimedia Appendix 2 [file mededu_v12i1e76924_app2.docx]

Step-by-Step Implementation Guide

This file is a Multimedia Appendix to a manuscript published in JMIR Medical Education.
For citation and copyright information, please refer to the following link:
<http://dx.doi.org/10.2196/76924>

This appendix provides the complete step-by-step implementation instructions for building the system described in the main text. Table 1 summarizes the platforms and credentials required; detailed procedures for each component follow below.

| Table 1. Platform URLs and credentials required for implementation | | | |
| --- | --- | --- | --- |
| Step in Tutorial | Service / Tool | Primary Action Required | Access URL |
| Prerequisites | OpenAI Platform | Get API Key: Sign up and generate a new secret key for the LLM. | <https://platform.openai.com/> |
|  | Anthropic Console | Get API Key: (Alternative) Sign up and generate a key if using Claude models. | <https://console.anthropic.com/> |
| Setting Up Dify | Dify Cloud | Create App: Log in to import the DSL file and configure the chatbot logic. | <https://cloud.dify.ai/> |
| Creating the Slack App | Slack API Console | Create App: Create a new Slack App, define Scopes, and get the xoxb token. | <https://api.slack.com/apps> |
| Configuring GAS | Google Apps Script | Deploy Script: Create a new project, paste the code, and deploy as Web App. | <https://script.google.com/> |

# Setting Up Dify

1. Create App: Log in to Dify and create a new app. Select "Chatflow" mode (not the basic Chatbot mode).
2. Import DSL: Click "Import DSL file" and upload Multimedia Appendix 2 (Dify DSL File). This reproduces the complete loop-based logic described in the main text.
3. Customize Configuration: Go to "Settings" → "Model Provider" and register your API Key (e.g., OpenAI, Anthropic).
4. Adjust Prompts: Modify the API model and meta-prompt content in the nodes as needed to suit your specific context.
5. Publish: "Publish" to save the current version.
6. Get Credentials: Open "API Access" in the left sidebar and copy the "API Key." Keep it safe, as it will be needed later when configuring Google Apps Script.

# Creating the Slack App

Once the AI processing engine has been configured in Dify, the next step is to create the bot account in Slack, which serves as the user-facing interface through which novice staff will interact with the system.

1. App Creation: In the Slack API Console, click "Create New App" → "From scratch." Name your app (e.g., "Rehab Feedback Bot") and select your workspace.
2. Setting Bot Token Scopes: Navigate to "OAuth & Permissions" in the sidebar, define the bot's privileges. Scopes act as keys to specific rooms; by default, a bot has no access and cannot perform actions. Educators need to explicitly grant it permission to perform actions like reading messages or sending replies. To maintain security while ensuring functionality, scroll down to "Bot Token Scopes" and add only the following necessary permissions:

- app_mentions:read (sees when the bot is mentioned)
- channels:history (reads messages in public channels)
- chat:write (sends messages)
- commands (uses slash commands)
- groups:history (reads messages in private channels)
- im:history (reads direct messages)
- mpim:history (reads group direct messages)
- users:read (reads user profiles)

1. Install to Workspace: Scroll to the top of the "OAuth & Permissions" page and click "Install to Workspace."
2. Copy Token: Copy the "Bot User OAuth Token" (starting with xoxb-...). This will be needed when configuring Google Apps Script.

# Configuring Google Apps Script

At this point, Dify handles the AI logic and Slack provides the chat interface, but the two platforms cannot communicate directly. Google Apps Script (GAS) serves as the bridge connecting them.

1. Create Project: Go to Google Apps Script and create a "New Project."
2. Copy Code: Delete any default code in the editor (function myFunction...) and copy-paste the entire script from Multimedia Appendix 3.
3. Set Admin ID: Locate the line const ADMIN_USER_ID = "..." at the top of the code. Replace "..." with your Slack User ID (e.g., U01234567) so the system sends error alerts to the administrator.
4. Configuring Script Properties (Secure Storage): To maintain security, never write API Keys directly in the code. Instead, store them in Script Properties, which act like a secure safe. To set this up, navigate to the "Project Settings" (gear icon) in the sidebar and scroll down to the "Script Properties" section. Then, click "Add script property" and register the following three items:

- DIFY_API_KEY: Paste the key from the Setting Up Dify section.
- SLACK_BOT_TOKEN: Paste the token from the Creating the Slack App section (xoxb-...).
- SPREADSHEET_ID: Create a new blank Google Sheet. Copy its ID (the long string of characters located between /d/ and /edit in the URL) and paste it here.
- Deploying as a Web App: Click the "Deploy" button in the top right corner and select "New deployment." In the configuration window, choose "Web App" as the type, set Execute as to "Me" and set who has access to "Anyone." This allows Slack to send messages to this script without authentication blocks. Click "Deploy" and copy the generated "Web App URL" for use in the next section.

# Final Configuration and Verification

With all three components in place, the final step is to link them together and verify that the system functions as intended.

1. Enable Event Subscriptions: In the Slack API Console, open "Event Subscriptions" and toggle "Enable Events" to On.
2. Paste URL: Paste the Web App URL (from the previous section) into the "Request URL" field and wait for it to show "Verified."
3. Subscribe to Events: Under the "Subscribe to bot events" section add: app_mention, message.channels, message.groups, message.im, and message.mpim.
4. Save Changes: Click "Save Changes." (Note: If prompted by a yellow banner asking users to "Reinstall App," follow the link).
5. Testing: In Slack, invite the bot (/invite @BotName) and send a test message (@BotName Hello, this is a test.). Success: The system is fully operational if the bot replies (via Dify) and the conversation is logged in your Google Sheet.

# Troubleshooting Common Issues

If the bot does not reply to the test message, follow this three-step diagnostic flow to identify the cause.

**Step 1: Check Dify Logs**

Open the Logs section in the Dify app.

Case A: Logs exist but show error or took a long time?

Diagnosis (The 60-Second Rule): Dify API calls timeout after 60 seconds. If the AI takes longer than this to generate a response, the connection terminates automatically.

Solution: Use faster models (like o4-mini) or shorten the instructions so responses are generated within 60 seconds.

Case B: NO logs found?

Diagnosis: The message never reached Dify. The issue lies in the GAS. Proceed to Step 2.

**Step 2: Check GAS Code and Properties**

Confirm that the script from Multimedia Appendix 3 was copied correctly.

Verify that all script properties (DIFY_API_KEY, etc.) are set correctly (check for accidental extra spaces at the beginning or end).

**Step 3: Check API Credits**

Confirm that billing is active with the LLM provider (e.g., OpenAI or Anthropic). If the payment method has expired or credits are exhausted, the API may silently refuse to generate a response.
