# Supplementary material for: Developing a Large Language Model–Based Feedback System for Case Report Writing in Rehabilitation Education: Tutorial
Source: JMIR Med Educ. 2026 Jun 15;12:e76924. doi: 10.2196/76924 (PMC13315997; doi:10.2196/76924)
Supplement: Multimedia Appendix 5 [file mededu_v12i1e76924_app5.docx]

**Detailed questionnaire results from the pilot study**

This file is a Multimedia Appendix to a manuscript published in JMIR Medical Education.
For citation and copyright information, please refer to the following link:
<http://dx.doi.org/10.2196/76924>

**Contents**

This supplementary file contains the detailed results of questionnaires administered before and after the implementation of the LLM-based feedback system.

- Page 1: Contents
- Page 2: Post-implementation survey results
- Page 3-5: Post-implementation survey results from instructors (Free Text Comments)
- Page 6-7: Post-implementation survey results from novice staff (Free Text Comments)

| **The Post-implementation survey results** | | | | |  |  |  |
| --- | --- | --- | --- | --- | --- | --- | --- |
|  |  |  |  |  | Instructors | | Novice staff |
|  |  |  |  |  | Median (Range) | | |
| 1. Survey on the Appropriateness of Feedback Content from LLM | | | | |  | |  |
| 1) Comparable to past human feedback? | | | | | 3 (2 to 4) | | 4 (4 to 5) |
| 2) Helpful for novice staff learning and growth? | | | | | 3 (1 to 4) | | 5 (4 to 5) |
|  | | | | |  | |  |
| 2. Survey on the Efficiency of Instruction | | | | |  | |  |
| 1) Did using this system save you time and reduce the number  of instruction sessions compared to traditional human-only feedback? | | | | | 4 (1 to 5) | |  |
| 2) Do you think using this system will reduce the burden on instructors  in the future? | | | | | 4 (1 to 5) | |  |
| 3) Do you think using this system will improve the learning efficiency  of novice staff in the future? | | | | | 3 (1 to 4) | |  |
|  | | | | |  | |  |
| 3. Survey on the System Usability Scale (SUS) | | | | |  | |  |
| 1) I think that I would like to use this system frequently | | | | |  | | 4 (4 to 5) |
| 2) I found the system unnecessarily complex | | | | |  | | 1 (1 to 2) |
| 3) I thought the system was easy to use | | | | |  | | 4 (4 to 5) |
| 4) I think that I would need technical support to use this system | | | | |  | | 3 (1 to 3) |
| 5) I found the various functions in this system were well integrated | | | | |  | | 4 (4 to 5) |
| 6) I thought there was too much inconsistency in this system | | | | |  | | 2 (1 to 2) |
| 7) I would imagine most people would learn to use this system quickly | | | | |  | | 5 (4 to 5) |
| 8) I found the system very cumbersome to use | | | | |  | | 1 (1 to 2) |
| 9) I felt very confident using the system | | | | |  | | 4 (3 to 5) |
| 10) I needed to learn a lot of things before I could get going with this system | | | | | | | 2 (1 to 3) |
| **The calculated SUS score** | | | | | | | **90 (70 to 95)** |
|  | | | | |  | |  |
| Explanation of question scores: 1: Strongly Disagree, 2: Disagree, 3: Neutral, 4: Agree, 5: Strongly Agree | | | | | | | |

| **The Post-implementation survey results from instructors (Free Text Comments)** | | | |
| --- | --- | --- | --- |
|  | Positive comments | Negative comments | |
| 1. Survey on the Quality of Feedback Content from LLM | -If used effectively, there would likely be fewer omissions in each content.  -Basic text is fine, but it seemed unable to express the flow of the story, especially the layered logical thinking.  -There are methodologies for constructing explanatory text, but sensitivity to words and the skill in handling words are not singular; in that sense, there is no single correct answer. Both machines and humans have their strengths and weaknesses.  -It seems that getting approval from AI is boosting confidence. I think this could potentially become a  driving force for overall progress. | -I felt that providing feedback on narrative content is difficult for AI.  -I think it was unable to fully interpret observed phenomena such as gait analysis. In that regard, it's necessary to delve deeper using the PDCA cycle based on hypotheses, so AI's knowledge and definitions alone are insufficient.  -It seemed that it wasn't being utilized to its full potential.  -While it's also influenced by the abilities and qualities of new staff, there was an overreliance on literature-based content, leading to a lack of focus on observing and thinking about actual patients.  -While this concern may become unnecessary if it becomes the norm in the near future, I worried that it might hinder the development of skills in writing one's own text and in mentoring juniors and students. | |
| 2. Survey on the Efficiency of Instruction | -By the time the report was submitted, the basic structure and writing had already been somewhat revised, allowing us to focus on providing guidance on more advanced aspects.  -I believe the efficiency of guidance improved because not only were the reports submitted with spelling errors, typos, and sentence structures already organized to some extent, but we were also able to review the Slack interactions before the final submission.  -The need for guidance on basic Japanese syntax has definitely decreased. However, it's highly likely that the individual abilities of the novice staff also play a role in this improvement.  -In recent years, whether due to changes in practicum guidelines or the impact of COVID-related restrictions on practical training, I feel that more novice staff members struggle with articulating their thoughts in writing compared to before. When submissions have insufficient grammar and structure, it's challenging to interpret them. With these points already addressed, I felt we could smoothly transition into providing feedback on clinical reasoning.  -The fact that feedback can be received even while at home  -The specific guidance on the text written by the novice staff seemed to help with their learning and presentation preparation. | -While there were no concerning issues with individual sentences, the overall impression was that of an immature writing style due to the lack of variety in conjunctions. It was necessary to suggest changing the conjunctions to better suit the context.  -Since the content produced by novice staff was initially disorganized, we first had them submit reports in bullet-point format. As a result, the efficiency of guidance remained unchanged from before.  -Setting aside the debate of whether it's good or bad, both instructors and novice staff felt that since AI judged it as passing, major revisions might not be necessary. This allowed them to complete the feedback within the designated time.  -The comments from the AI often aimed to add more information to make the details clearer. However, since we specifically asked for focused and summarized reports, it seemed that the AI's comments were not utilized much.  -Ultimately, it is up to the user. Some people may improve their learning efficiency by using it, while others might mistakenly believe they are improving.  -It was unclear whether the new staff understood how to use it effectively.  -The basic thoughts and considerations in the submitted assignments were fine, but it seemed they were overly influenced by the feedback content. | |
| 3. Did the use of this system change the content of the feedback you provide? | -In terms of summarizing content, it was helpful in some aspects of how to explain things.  -I was able to focus on providing specialized feedback.  -It seems that efficiency improved and time was saved, allowing for more thorough consideration of the presentation content.  -Basically, I was able to focus on providing specialized feedback. | -Compared to previous novice staff training, I don't think there's much difference at this point.  -It may have been a hindrance in encouraging the thinking process of observing and considering the patient. | |
| 4. Are there any areas where the system needs improvement? | -I am humbled. Is it the richness and difficulty of the freedom in Japanese expressions?  -More than the system itself, I felt that it is necessary to not only explain at the beginning but also to discuss and understand how to use it along the way.  -Among the comments from the AI, there were some that praised the writing unnecessarily. I felt that if the AI approves but the instructor thinks it should be corrected, the beginner staff might get confused.  - I think there could be more feedback that encourages thinking. | | |
| Abbreviations: LLM, Large-scale Language Models; | | |  |

| **The Post-implementation survey results from novice staff (Free Text Comments)** | | | |
| --- | --- | --- | --- |
|  | Positive comments | Negative comments | |
| 1. Survey on the Quality of Feedback Content from LLM | -The assistance with text structure was invaluable, especially for someone like me who struggles with writing.  -It was helpful that you pointed out areas needing improvement or additions.  -I received feedback on detailed aspects like interpretation and reasoning.  -Clearly indicating strengths and areas for improvement was useful.  - I found it helpful in summarizing reports in fields where I have limited knowledge, as it provides expert opinions based on specialized knowledge.  -For novice staff who lack specialized knowledge, I found it particularly useful in helping them articulate their thoughts into written form, even when their ideas are clear in their minds but difficult to express in writing.  -Responses come back quickly.  -The feedback is documented, so it's not forgotten.  -It reduces mental stress.  -It's beneficial because detailed feedback is provided, even on minor points. | -I appreciated the additional advice. However, more specific expert opinions would have been beneficial. | |
| 2. Are there any areas where the system needs improvement? | -I thought that if the feedback could provide more insightful opinions, it would result in an even more robust report.  -I felt it was a bit difficult to submit casually since everyone can see it. I think it would be more user-friendly if we could use it easily and immediately after organizing our thoughts.  -I think it would be more readable if there were underlines or similar markings just for the points that need improvement. | | |
| Abbreviations: LLM, Large-scale Language Models; | | |  |
